# Supplementary material for: Targeting phosphorus loss with carbon farming practices? Results from an on‐farm study
Source: J Environ Qual. 2026 Apr 29;55:e70190. doi: 10.1002/jeq2.70190 (PMC13129245; doi:10.1002/jeq2.70190)
Supplement: Supplementary file 1 — Supporting Information [file JEQ2-55-0-s001.docx]

Supplementary information: graphs on P balance and soil water conditions in the carbon farming and control fields arranged by carbon farming practice.


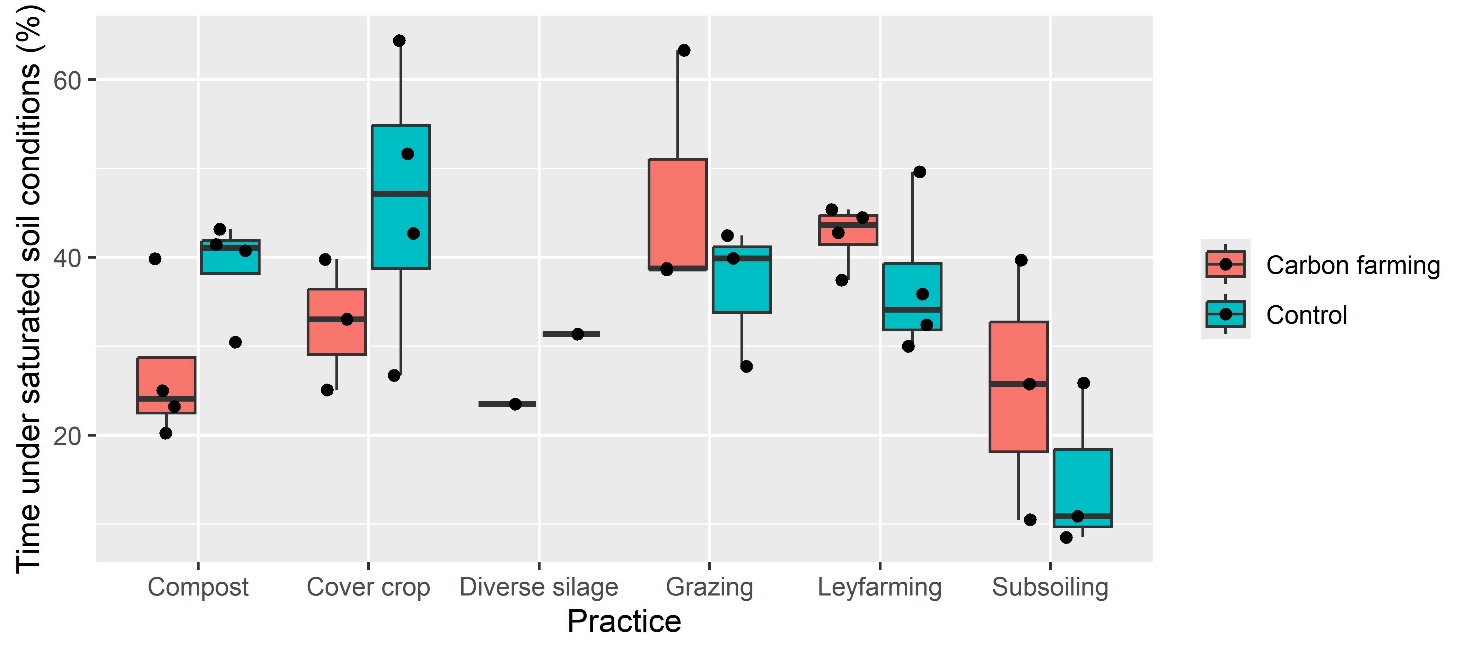


Figure S1. The effect of carbon farming practices on soil moisture conditions was variable and depended on the carbon farming practice.


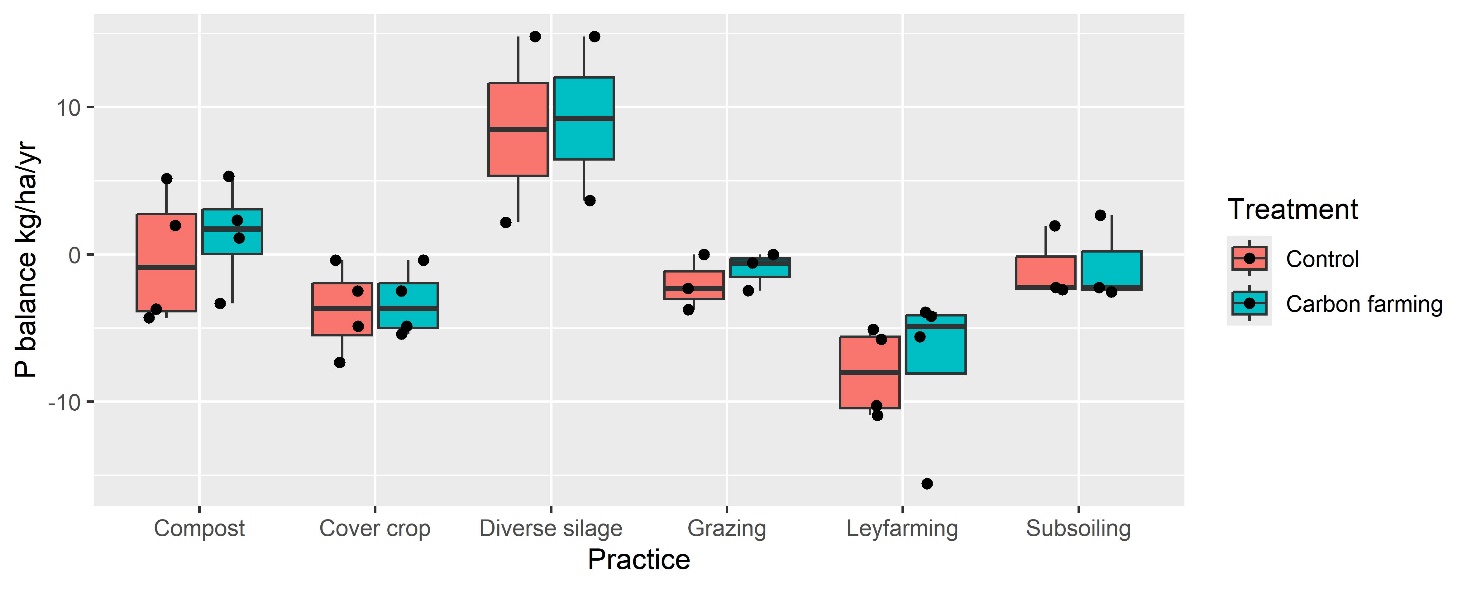


Figure S2. The effect of carbon farming practices on the P balance was variable and depended on the carbon farming practice. Most notable effects were found for leyfarming, grazing and compost application, which involved larger amounts of manure or compost application than the control plots.
